# Structure–Function Relationship and Physiological Roles of Transient Receptor Potential Canonical (TRPC) 4 and 5 Channels

**DOI:** 10.3390/cells9010073

**Published:** 2019-12-27

**Authors:** Jinsung Kim, Juyeon Ko, Chansik Hong, Insuk So

**Affiliations:** 1 Department of Physiology, College of Medicine, Seoul National University, Seoul 03080, Korea; jinsung_kim@snu.ac.kr (J.K.); juuyeon@snu.ac.kr (J.K.); 2 Department of Physiology, College of Medicine, Chosun University, Gwangju 61452, Korea; cshong@chosun.ac.kr

**Keywords:** structure–function relationship, transient receptor potential canonical, TRPC

## Abstract

The study of the structure–function relationship of ion channels has been one of the most challenging goals in contemporary physiology. Revelation of the three-dimensional (3D) structure of ion channels has facilitated our understanding of many of the submolecular mechanisms inside ion channels, such as selective permeability, voltage dependency, agonist binding, and inter-subunit multimerization. Identifying the structure–function relationship of the ion channels is clinically important as well since only such knowledge can imbue potential therapeutics with practical possibilities. In a sense, recent advances in the understanding of the structure–relationship of transient receptor potential canonical (TRPC) channels look promising since human TRPC channels are calcium-permeable, non-selective cation channels expressed in many tissues such as the gastrointestinal (GI) tract, kidney, heart, vasculature, and brain. TRPC channels are known to regulate GI contractility and motility, pulmonary hypertension, right ventricular hypertrophy, podocyte injury, seizure, fear, anxiety-like behavior, and many others. In this article, we tried to elaborate recent findings of Cryo-EM (cryogenic-electron microscopy) based structural information of TRPC 4 and 5 channels and domain-specific functions of the channel, such as G-protein mediated activation mechanism, extracellular modification of the channel, homo/hetero-tetramerization, and pharmacological gating mechanisms.

## 1. Introduction

Transient receptor potential canonical (TRPC) channels constitute non-selective, Ca^2+^-permeable channels in mammalian cells [1,2,3]. There are 7 TRPC subtypes, namely, TRPC1–7, where 6 subtypes except for TRPC2 can be found in mammalian cells [4]. Based on amino acid sequence similarities, TRPC1, 4, and 5 channels are grouped as one subfamily and TRPC 3, 6, 7 are grouped as the other [5,6]. The expression pattern of TRPC1 in mammalian tissue is ubiquitous. TRPC4 and 5, however, show somewhat localized expression profile such as brain [7,8,9,10,11,12,13,14,15,16,17,18], gastrointestinal tract (GI tract) [2,3,19,20,21,22], ovary [23,24], endometrium [25,26], ventricular myocytes [27,28,29], and vasculature [30,31,32,33]. In such tissues, TRPC 1, 4, and 5 channels are known to modulate fear or stress response, integrity of dopaminergic motor neurons such as substantia nigra, hypertrophic cardiomyopathy, endothelial dysfunction, cholinergic contraction of GI smooth muscle, and many others. Because of the overlapping expression pattern and capability for heteromerization, channel composition in the plasma membrane of native tissues is usually heterogeneous; TRPC4/4, TRPC5/5, TRPC1/4 or TRPC1/5 channels can all be found.

Classically, TRPC4 and 5 channels are known to be activated by G-proteins and their downstream signal transduction pathways, such as Gq-PLC pathway [2,3,21,22,34,35]. Intensive researches have been done on delineating functional relationship among TRPC4, 5 channels and phosphatidylinositol 4,5-bisphosphate (PIP_2_) [36,37,38,39,40,41,42], intracellular Ca^2+^ ([Ca^2+^]_i_) and protein kinase C (PKC) [43,44]. Recently, it was suggested that not only downstream signal transduction pathways but active form of alpha subunits of G-proteins (Gαi or Gαq) per se can activate TRPC4, TRPC5, TRPC1/4, or TRPC1/5 channels as well [38,39,45,46]. Earlier studies of TRPC channels have gone through some struggles due to lack of channel-specific agonists or antagonists. In a sense, a recent development of channel-specific pharmacological tools such as ML-204 [47], (-)-Englerin-A (or simply, Englerin-A) [48] and Pico-145 [49] has brought great utility to the TRPC community. With such pharmacological treasures, dissecting each contribution of the TRPC channels among the mixture of channels in plasma membrane is becoming clearer than ever. In spite of functional utility with which the channel-specific drugs invest, however, study for mechanism of action of those drugs with structural insight is unfortunately limited.

The great technological advance in the Cryo-EM ‘resolution-revolution’ has helped in revealing atomic structures [50] of many transmembrane proteins, not to mention TRPC channels [51,52,53]. Meanwhile, there has been more than 30 years of discussion about functionality of the TRPC channels. Possible molecular candidates that might affect opening or closing of the channel had been numerously reported, and the domains or amino acids within the channel that might be responsible for the interaction with such molecular candidates have been rigorously found.

In this article, we first tried to reorganize some critical results in functional studies of TRPC1, 4, or 5 channels based on our expertise. As a next step, we elaborated a possible structure–function relationship in each topic based on the Cryo-EM structure of the channels. Finally, we conclude this article with challenging questions that still have not been answered even with the Cryo-EM structure. We presume that such questions should be answered in order to fully understand TRPC4, 5, and related heterotetrameric channels (TRPC1/4 and TRPC1/5). 

## 2. General Architecture of TRPC4 and TRPC5 Channels

**Figure 1:**
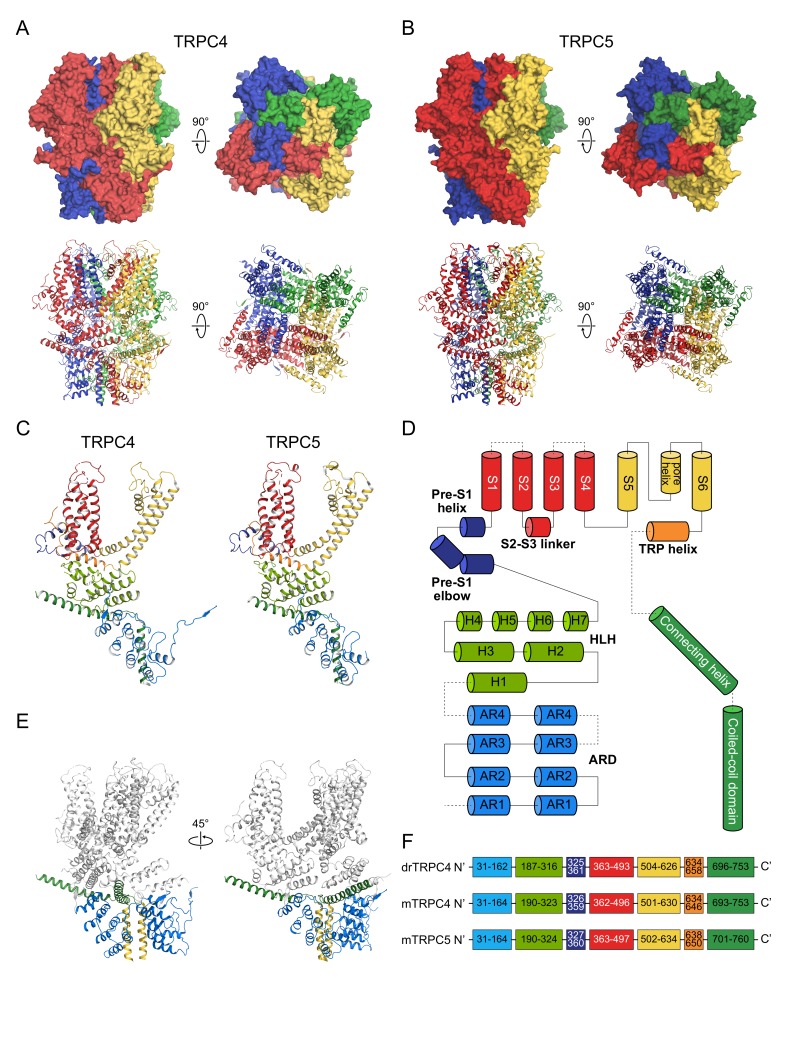
General architecture of the TRPC4 and TRPC5 channels. (**A**,**B**) Tetrameric structures of the channels. Upper panel: surface map of the channels; *Lower panel*: cartoon diagram of the channels. (**C**) Monomers of each channel. The colors of each domain correspond to the domain diagram in (**D**,**F**). (**D**) Domain diagram of TRPC4 and 5 channels. From the *N*-terminal cytoplasmic domain to the C-terminal cytoplasmic domain, ARD, HLH, Pre-S1 elbow, Pre-S1 helix, VSLD, S5, S5-S6 linker, pore helix, S6, TRP helix, connecting helix, and the coiled-coil domain are shown. (**E**) Relationship between ARD and the connecting helix. Blue domains: ARD; Green domains: connecting-helix; Yellow domains: coiled-coil domains. (**F**) Amino-acid numbers corresponding to each domain.

Like many of voltage-dependent ion channels, TRPC channels share a common architectural framework of such channels. TRPC channels have six transmembrane segments and two large cytoplasmic domains, namely, the N-terminal domain and C-terminal domain. At N-terminus, there are 4 ankyrin-repeat domains (ARD) which have crucial roles in subunit–subunit multimerization, membrane trafficking, and proper protein folding. Between ARD and the first transmembrane segment (S1), there exists a helical domain whose axis is somewhat parallel to the inner border of the plasma membrane: ‘pre-S1 helix’. Between the pre-S1 helix and ARD, 7 α-helices connected with loops can be found. These are called the ‘helix-loop-helix (HLH)’ domain. The last component in the N-terminal cytoplasmic domains is the ‘pre-S1 elbow’ which is between the HLH and pre-S1 helix. After the pre-S1 helix, four transmembrane segments (S1-S4) constitute the voltage-sensor like domain (VSLD) of the TRPC channels whose functional role and topology resemble the voltage-sensor domain of the voltage-gated potassium channels [54]. The VSLD of TRPC4 and 5 channels share great similarity with the VSLD of TRPC3 or TRPC6 channels except S3 [55,56]. S3 of the TRPC3 and TRPC6 channels are longer than S3 of the TRPC4 and TRPC5 channels, protruding beyond the extracellular borderline of the plasma membrane. The difference in S3 may reflect some possible discrepancies in extracellular channel modulation mechanisms between TRPC3, 6, and TRPC4, and 5 channels. The fifth and sixth transmembrane segment (S5, S6), the extracellular linker sequence between S5 and S6 (S5-S6 linker) and helical structure within the S5-S6 linker (‘pore helix’), constitute the core framework for the central ion conduction pathway in a domain-swapped fashion. After S6, a long C-terminal cytoplasmic domain unfolds where a hydrophobic helical domain comes first. This hydrophobic helical domain has a TRP signature sequence (EWKFAR), hence called ‘TRP helix’ or ‘TRP box’. The TRP sequence can be found not only in TRPC channels but in the TRPM and TRPV superfamily. It is generally known that a mutation in the TRP sequence could cause drastic effect in the channel function. In the TRPV channel, for instance, it has been reported that indole nitrogen in tryptophan makes strong hydrogen bonds with carbonyl oxygen in the S4-S5 linker sequence. Mutation of tryptophan resulted in the gain-of-function mutant of the TRPV3 (W692G) and TRPV4 (W773R) channels [57,58]. The effect may be partly attributable to structural proximity among the TRP helix, S4-S5 linker, and pre-S1 elbow. The next helical structure runs diagonally from the channel periphery towards the inner vestibule. This helical structure is called the ‘rib-helix’ or ‘connecting helix’ and is located distal to the TRP helix. The structure between the TRP helix and the connecting helix is missing in the Cryo-EM structure of both TRPC4 and TRPC5. The connecting helix of one subunit penetrates the space between the ARD of the subunit and ARD of the adjacent subunit (Figure 1). Hence it is generally conceived that the connecting-helix may have an important role in inter-subunit interaction. After the connecting helix, a longitudinal ‘coiled-coil helix’ or ‘coiled-coil domain’ follows. 

Based on topology, it is presumed that there are further cytoplasmic domains distal to the coiled-coil helix, which in this article we call ‘far south domains (FSD)’. Moreover, many of the key domains or amino acids responsible for channel tetramerization, G-protein mediated gating, or membrane trafficking, are located in the FSD. We will discuss them in detail later in the article.

## 3. Ion Conduction Pathway, Selectivity, and Rectification

### 3.1. Conduction Pathway and Selectivity

A pivotal study describing conduction pathway of the TRPC4 and TRPC5 channels was done in embryonic brain and human embryonic kidney (HEK) 293 cells stably expressing human muscarinic receptor subtype 1 (HM1 cell) [15]. When TRPC5 was over-expressed in the HM1 cell, they elicited large, double-rectifying cationic current in response to extracellular 100 μM carbachol, a potent acetylcholine analogue. However, mutant TRPC5 channel whose LFW moiety at 575–577th position was changed to alanine (TRPC5^LFW/AAA^) failed to elicit any of cationic current in response to muscarinic stimulation. The mutant had no defect in trafficking to plasma membrane, hence suggested as a pore-collapsed form of the channel. Moreover, co-expression of TRPC5^LFW/AAA^ with wild-type channel severely reduced whole-cell current of TRPC4 and TRPC5 but not TRPC3, suggesting that the TRPC4 and TRPC5 channels can share pore architecture in tetrameric assembly, whereas TRPC3 cannot. 

About 10 years later, similar results were observed [59]. In HEK293 cells expressing muscarinic receptor subtype 3 (mAChR_3_), over-expression of both TRPC1 and TRPC4 yielded outward-rectifying cationic current of heteromeric TRPC1/4 channel in response to 100 μM carbachol. When LFW moiety of the TRPC1 channel was changed to alanine, the whole-cell current of the TRPC1/4 channel was abolished. Although the pore may have been collapsed, the TRPC1^LFW/AAA^ channel retained its capacity for subunit assembly. Fӧster resonance energy transfer (FRET) signal from TRPC1^LFW/AAA^-CFP and TRPC4-YFP was intact in plasma membrane. 

Cryo-EM structure of TRPC4 and TRPC5 locate the LFW moiety of both channels at pore-helix. At the pore helix, delocalized electrons of π system of aromatic rings of phenylalanine and tryptophan in LFW moiety seem to have π–π interaction with each other and stabilize the entire pore structure [51,52]. The structures also show that from the selectivity filter to inner gate, arginine, glycine, isoleucine, arginine, and glutamine may play significant roles in the conduction pathway. In TRPC5, mutations targeted for those residues abolished current response to extracellular lanthanides (Gd^3+^). The mutants showed diverse results in response to extracellular Englerin-A. N584A showed only a minute amount of the current which is much smaller than wild-type. G581A showed large current but fast inactivation with near zero steady-state conductance was followed. Mutating three amino acids near the putative inner gate (I621A/N625A/Q629A) elicited large current and slow inactivation was observed [51] (Table 1). 

**Figure 2:**
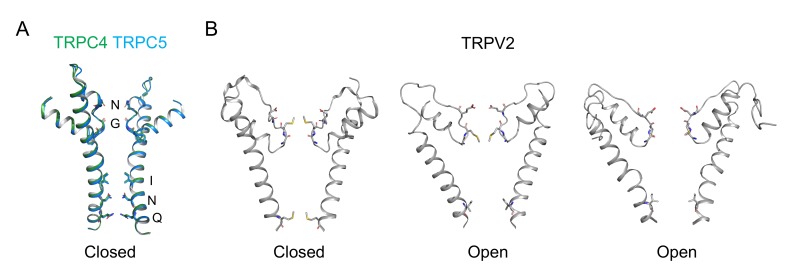
Putative pore models of TRPC4 and 5 channels based on conformation changes shown in TRPV2 channel. (**A**) Pore structure of TRPC4 (green) and TRPC5 (blue) channels. (**B**) Pore structure of TRPV2 channels. *Left panel*: closed state; *Middle panel*: open state with widened lower, helix-bundle-cross (HBC) gate; *Right panel*: open state with widened selectivity filter gate (SF gate) and HBC gate.

Since both Cryo-EM structures of TRPC4 and TRPC5 show closed state, it is still not clear how conducting ions interact with amino acids within conduction pathway. Likewise, it is still hard to explain which residues or cooperation of such residues are responsible for non-selectivity to cations, namely, permeability ratios such as P_Cs_/P_Na_ or P_Ca_/P_Na_. If we are to make a model based on conduction pathways of already-published structures such as TRPV channels [60,61], TRPC may have two independent gates governing conduction pathway. One may locate near the selectivity filter (upper gate) and the other may locate near the cytoplasmic end of the conduction pathway (lower gate) (Figure 2).

### 3.2. Rectification

One of the most fascinating features of TRPC4 and TRPC5 channels is the shape of I-V curve [34]. In physiological membrane potential (−90 mV to 40 mV), the I-V curves of both channels mimic the inward rectifier, such as inwardly-rectifying potassium channels. When the membrane potential is depolarized more than 40 mV, both channels elicit sudden burst of outward current. This unique feature of I-V curve is often called double-rectifying shape. Extremely low conductance in membrane potential range of 0 mV to 40 mV is a characteristic feature of the channel and can be used as an electrophysiological index to identify whether a measured current across the membrane is due to TRPC4, 5 channels or not. The low conductance at depolarized potential (0 mV to 40 mV) of TRPC4, 5 channels is as important as an experimental index but so much as a physiological implication. It is well known that TRPC4 or 5 channels show membrane excitatory effect. This may be due to their non-selectivity, near-zero reversal potential and calcium permeability. But most of all, an inwardly-rectifying I-V curve takes great part. TRPC4 or 5 channels, a mild membrane depolarizer, can efficiently clamp the membrane potential at near zero because the channel permits no outward current at positive membrane potential. Conversely, had TRPC4 or TRPC5 channels somewhat lost their inwardly-rectifying manner in physiological potential range and become an outward-rectifier, their role would have been changed to membrane stabilizers rather than depolarizers. This sudden change can be achieved whenever TRPC1 is incorporated to TRPC4 or TRPC5 channels. In other words, heteromeric TRPC1/4 or TRPC1/5 channels show outwardly-rectifying I-V curves. An extensive review regarding electrophysiological difference between homomeric and heteromeric channels can be found elsewhere [2,3,34,62]. 

One of the earliest studies demonstrating molecular mechanism of inward rectification of TRPC5 channel showed that intracellular magnesium ([Mg^2+^]_i_) blocks the channel at depolarized potential, by binding aspartate residue D633 of the channel [63]. Mutation of D633 to asparagine abolished inward-rectification feature of the channel in single channel conductance level. It is also worthy to remark that mutation to D633N not only changed I-V shape but critically diminished absolute amount of inward current through the channel. These results have suggested that the aspartate residue is crucial for crucial for magnesium binding and inward-rectification, and the stabilization of the residue by electrostatic interaction with Mg^2+^ seems to be necessary for the channel to elicit inward cationic current. A similar mechanism was also observed in inwardly rectifying potassium channel where [Mg^2+^]_i_ [64,65,66] and intracellular polyamines [67,68,69] such as spermine and spermidine block the cytoplasmic entrance of the pore and literally plug it at depolarized potential. The studies of inwardly rectifying potassium channels have demonstrated that intracellular polyamines can make stable electrostatic interaction with negatively charged amino acids within the pore since polyamines are polycationic molecules with 2+~4+ valences in physiological pH [70]. 

Interestingly, there had been studies suggesting that polyamines could modulate the activity of TRPC4 channels. In guinea pig ileal myocytes, Tsvilovskyy et al. showed that cationic current evoked by muscarinic stimulation (mIcat) was inhibited by extracellular polyamine, especially spermine [71]. The inhibition was not voltage-dependent and I-V curve retained doubly-rectifying shape. Moreover, they also mentioned that concentration of extracellular polyamines in GI tract was in mM range and intracellular concentration of polyamines was in μM level. Later, two pivotal studies showed that molecular candidate for mIcat in ileal myocyte is TRPC4 and TRPC6 channels and that cholinergic activation, namely, muscarinic receptor subtype 2 and 3 are governing the channel function in the tissue [20,22].

**Figure 3:**
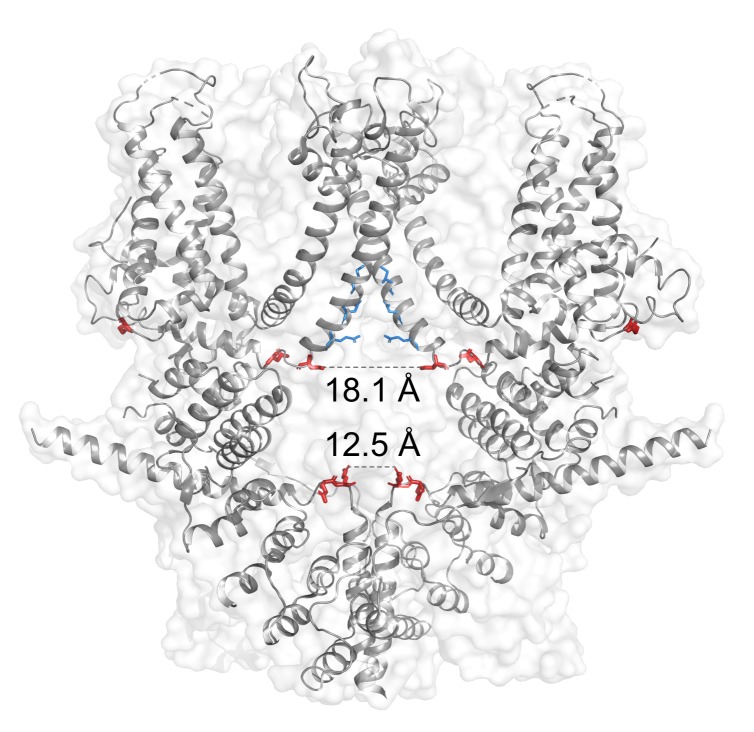
(*Red sticks*) Residues responsible for Mg^2+^ and spermine-mediated inward-rectification of TRPC4. Distances in Angstrom between D629 residues (18.1) and E728 residues (12.5) are shown. (*Blue sticks*) Residues responsible for putative inner gate of the channel: I617, N621, and Q625.

The effect of intracellular polyamine to TRPC4 channel was verified later. With mutant TRPC4 channels, Kim et al. showed that glutamate residues at 728th and 729th position are possible binding sites for intracellular spermine [72]. Intracellular spermine reduced outward current dramatically, leaving inward current less changed. This asymmetrical current inhibition suggested voltage-dependency of TRPC4 channel block by intracellular spermine. The claim was supported by the left shift of conductance curve of TRPC4 channels. A different study with similar experimental setup claimed that there exist additional binding sites for spermine [73]. In this study, the effects of D629A mutation have been addressed in TRPC4 channels. D629 is equivalent site to D633 of TRPC5, hence may be responsible for Mg^2+^ block and channel stabilization. Like TRPC5, D629A mutation reduced absolute current of TRPC4 channel. Moreover, spermine could not block D629A channel as efficiently as wild-type, suggesting that D629 may be involved in spermine binding. All residues deemed to be involved in spermine-mediated inward-rectification are highlighted (Figure 3) based on Cryo-EM structure of TRPC4 channel. Most of the residues found in functional studies circumference inner vestibule and lower border of inner gate, except for the two: E648 and E649. These residues locate right after TRP helix and have spatial proximity to pre-S1 elbow. These residues may attract cytosolic spermine and increase the local concentration of spermine near the channel periphery. Overall effect of E648 and E649 would be reducing access resistance for the following reaction:

[Spermine]_cytosol_ → [Spermine]_inner vestibule_ (1)

In a sense, it seems quite surprising that intracellular spermine cannot affect heteromeric channels, i.e., TRPC1/4 and TRPC1/5 channels [72]. Since the I-V curve of heteromeric channels is outwardly-rectifying, intracellular spermine may strongly reduce the outward current if they can exert similar cytoplasmic blocking effect to the heteromeric channel. The stark difference between homomeric and heteromeric channel may be attributed to different structure of two channels, especially c-terminal domain and inner vestibule. 

In summary, the studies of TRPC5 and TRPC4 emphasize that amino acids constituting inner vestibule are crucial for inward-rectification of the channel. Intracellular magnesium bind to a aspartate residue which exist in close proximity with one another in tetrameric formation and the attractive electrostatic interaction between magnesium and aspartate is necessary to elicit stable cationic current through the channel. Acidic amino acids in 720–740 region of TRPC4 channels are crucial for spermine-mediated inward-rectification. 

## 4. Homo- and Hetero-Tetramerization

### 4.1. General Aspect of Homo- and Hetero-Tetramerization

As described in the Introduction, TRPC1 can be found in almost every tissue in mammalians whereas expression of TRPC4 or TRPC5 is rather specific. Given that the expression of each channel subunits is tightly regulated, there exists nonzero chance that population of channels composed of TRPC subunits is heterogeneous. In other words, all the results of possible combinations of subunit assembly can be observed in a plasma membrane of a single cell. For instance, if a smooth muscle cell in ileum expresses both TRPC1 and TRPC4, both the heteromeric TRPC1/4 channel and homomeric TRPC4/4 channel could be found in plasma membrane. 

One of the earliest studies addressing the issue was done in HEK293 and Chinese hamster ovary (CHO)-K1 cells using over-expression system of TRPC1, 3, 4, 5, and 6 channels [74]. Co-expression of dominant-negative form of TRPC6, i.e., pore-collapsed form of TRPC6 (TRPC6^LFW/AAA^) abolished TRPC3 current but not TRPC4 and TRPC5 current. The study also designed fluorescence protein tagged channels of which CFP or YFP tags are linked at C-terminus of the channel. Using such constructs, the study evaluated which combination is most feasible. As a result, TRPC1, 4, and 5 showed strong FRET signal with one another. TRPC3 and 6 also showed high FRET signal. In contrast, no FRET signal was observed between two groups. Co-immunoprecipitation (Co-IP) yielded similar results. As mentioned earlier, heteromerization between TRPC1, 4, and 5 was also observed in embryonic brain [15]. A recent study demonstrating neuro-protective effect of TRPC1 also showed that both TRPC1 and TRPC5 are expressed in substantia nigra and heteromeric population (TRPC1/5) is substantial in normal tissue [13]. They have argued that excessive Ca^2+^ influx through homomeric TRPC5 channel is the key pathologic step in Huntington’s disease and the pathologic Ca^2+^ influx is triggered by reduced expression of TRPC1, hence increased portion of homomeric TRPC5 in plasma membrane. 

Based on nearly 20 years of cumulative results, it is now well acknowledged that all TRPC1, 4, and 5 subunits contribute equally to form a conducting pore in a tetrameric assembly. In a sense, the difference in major channel characteristics such as activation mechanism, selective permeability, pharmacological sensitivity, and membrane excitatory effect among TRPC1/4, TRPC1/5, TRPC4/4, and TRPC5/5 channels could not be ignored. Truly, the strength of electric field exerted by amino acids in selectivity filter was starkly different between homomeric TRPC4/4 channels and heteromeric TRPC1/4 channels. Namely, the Eisenmann sequence, an estimate for pore field strength, was different between two channels. Homomeric channels showed weak pore-field strength while heteromeric channel showed strong pore-field strength [59,75]. 

### 4.2. Molecular Mechanism of Tetramerization Process

The studies above are milestone of the endeavor for understanding the tetramerization process of TRPC channels. Information given by the studies such as the existence of heteromeric TRPC channels in plasma membrane and an empirical proof for combinatorial number of cases all helped to deepen our understanding of the topic. Despite of those elegant studies, however, the molecular mechanism of tetramerization was still unknown. 

In the late 2000s, molecular mechanisms of tetramerization among TRPC4 and TRPC5 channels were addressed from an Austrian group and a Canadian group. Using FRET method and size-exclusion chromatography, they suggested that a part of 1st ARD (69–98) is crucial for homotetramerization process of TRPC5 [76], and that parts of 3rd and 4th ARD (87–172) and *N*-terminus coiled-coil domain (254–304) are important in the homotetramerization process of TRPC4 [77]. Their arguments were based on the channel topology at the time. Up-to-date domains are 2nd ARD (69–98) of TRPC5, 2nd and 3rd ARD (87–172), and helix-loop-helix (HLH) domain (254–304) of TRPC4. Based on domain-specific results, one study postulated two possible models for the homotetramerization process of TRPC4 channels. The first model assumed that each subunit has full capacity for tetramerization and no further constraints were necessary. The second model assumed that two monomers should form dimer first, generating two dimers in total. This initial dimerization process is achieved using 3rd ARD (87–172) according to the model. Then, two dimers are assembled together to form a tetramer by interactions of HLH domains (254–304). A recent study suggested similar results using FRET method and thorough electrophysiology. With a number of *N*-terminal truncation mutants of TRPC4 channels, the study argued that 98–124 residues in *N*-terminus (3rd ARD) and 700–728 residues in the C-terminus (connecting-helix) play a great role in the homotetramerization process of TRPC4 [78].

In theory, the large quaternary structure of TRPC channels would require delicate post-translational modification in various intracellular organelles, especially in terms of tetrameric assembly. Therefore, finding out the time and the location of the tetramerization process would be both intriguing and necessary. Recently, there has been a report that tetramerization process may occur in endoplasmic reticulum (ER)-Golgi system [79]. When 553rd cysteine residue of TRPC5 was mutated to serine or alanine, a membrane translocation was severely damaged. Expression level of TRPC5 channel at plasma membrane was significantly low in TRPC5^C553S^ mutant compared to wild-type (WT) channel. Moreover, co-expression of TRPC5^C553S^ mutant even reduced surface expression level of wild-type channel. The most interesting part of the study is that high FRET signal was detected inside Golgi complex when TRPC5^C553S^-CFP and TRPC5^WT^-YFP were co-expressed. Based on the results, the study claimed that C553 is crucial for membrane translocation process of TRPC5 and the tetramerization process may occur at or before Golgi.

The molecular mechanistic study for heteromerization process is unfortunately limited despite of its significance. One study so far represented specific domains responsible for heteromerization process of TRPC1/4 and TRPC1/5 channels. Using FRET method and electrophysiological recording subjected to various truncation mutants, Myeong et al. suggested that 700–728 residue (connecting-helix) of TRPC4 and 707–735 residue (connecting-helix) of TRPC5 are important in heteromerization with TRPC1 [80]. It is surprising, however, that TRPC1 utilizes different domains for TRPC4 and TRPC5. For TRPC1/4, 725–745 region of TRPC1 is used as an inter-subunit interface, while the 673–725 region is used for TRPC1/5. Topologic analysis based on sequence alignment and Cryo-EM structure of TRPC4 and TRPC5 suggests that both regions of TRPC1 correspond to putative connecting-helix of TRPC1 channel. 

**Figure 4:**
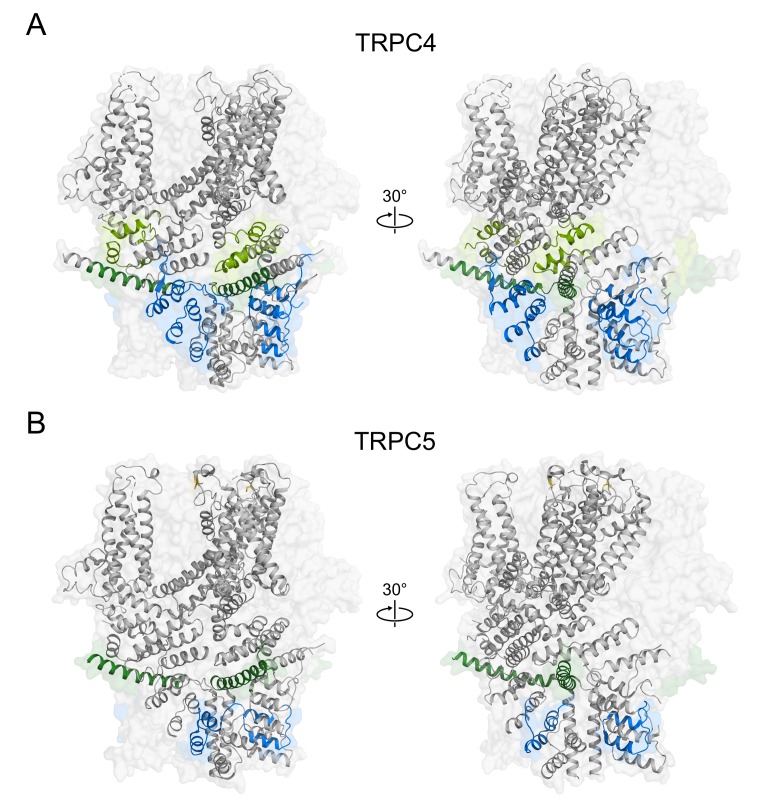
Domains responsible for tetramerization process in TRPC4 and TRPC5 channels. (**A**) Domains in TRPC4 channels. *Blue domains*: ARD; *Light green domains*: HLH; *Green domains*: connecting helix. (**B**) Domains in TRPC5 channels. *Blue domains*: ARD; *Green domains*: connecting-helix.

In summary, both N-terminal and C-terminal cytoplasmic domains seem to have crucial role in tetramerization process among TRPC1, 4, and 5 channels. As mentioned earlier in general architecture, ARDs and connecting-helices are of special interest. Last but not least, cysteine residue in the S5-S6 linker seems to have a great role in tetramerization process (Figure 4, Table 2 and Table 3). Indeed, two cysteines in S5-S6 linker, C549/C554 in TRPC4 and C553/C558 in TRPC5, bear many complicated functions of which we will discuss in the following section.

## 5. Cysteine Modification

### 5.1. Functional Studies of Cysteine Modificaiton

The thiol group (-SH) of cysteine grants chemical flexibility to the protein. Free thiol group can undergo numerous chemical reactions and the reaction pathway varies according to the type of reactant partner, redox potential of reaction compartment, and many others. One of the most studied reaction of thiol group in a biological system would be formation and dissociation of disulfide bond between two cysteine residues. It is generally accepted that the first formation of disulfide bridge of a protein occurs inside ER [81,82]. Since both intracellular and extracellular fluid can contain diverse enzymes or biomolecules for rigorous redox reaction, however, a preformed disulfide bridge can be broken at certain condition. Likewise, free thiol groups can be oxidized by cellular oxidants such as glutathione and form disulfide bridge outside ER. 

Among number of cysteine residues in TRPC channels, two cysteines (C549/C554 of TRPC4 and C553/C558 of TRPC5) have been studied most intensively. The cysteine residues are conserved in TRPC1, 4, and 5 channels but not in TRPC3 and 6 channels [83]. The earliest study was about S-nitrosylation of cysteine residues of TRPC5 channel and the outcome of such reaction onto the channel activity [83]. Yoshida et al. showed that increasing NO concentration using NO-donating chemical (*S*-nitroso-*N*-acetyl-dl-penicillamine, SNAP) induced robust TRPC5 activity [83]. The effect was independent from cGMP pathways suggesting that increased cytosolic NO may directly interact with channel protein. A similar effect was observed when a chemical, which can undergo S-nitrosylation reaction with thiol group (5-nitro-2-PDS), was applied. Using cell-impermeable chemicals, they also suggested that such S-nitrosylation reaction may occur through a route from cytosolic space to the free thiol residue of the channel. In conclusion, the study claimed that S-nitrosylation at C553 could result in the opening of the channel via cytosolic approach and that channel closes when intracellular reducing agent reduces cysteine residue to –SH. Along with the results above, the study also showed that despite of their irresponsiveness to *S*-nitrosylation C553S and C558S can be activated by conventional activation mechanisms such as muscarinic stimulation. Moreover, the surface level of C553S and C558S mutant was indifferent from wild-type TRPC5 channel, suggesting that two cysteine residues rarely affect membrane translocation of the channel. Two years later, another study reported that reduction of cysteine residues by extracellular reducing agent such as dithiothreitol (DTT) activates the TRPC5 channel and that C553 and C558 are target sites for reduction reaction [84]. According to the study, TRPC5 gains activity when C553 or C558 has free thiol group rather than oxidized form. Another comprehensive study for C553 and C558 came out about 10 years later. In this article, Hong et al. showed that C553S, C558S, and C553S/C558S mutants are all irresponsive to muscarinic stimulation, extracellular lanthanides, and reducing agent (DTT) [79]. The study suggested that cysteine residues are involved in tetramerization process and membrane translocation process of TRPC5 channels. In view of whole-cell current of such mutants, findings suggested by one of the latest studies in the topic looks surprising. Jeong et al. showed that C553S and C558S mutant TRPC5 channels could not elicit cationic current in response to extracellular Englerin-A [85]. However, double mutant of both cysteines (C553S/C558S) elicited large Englerin-A-mediated current.

As we have seen through, accumulated results of C553 and C558 show some inconsistencies among one another. The current response to reducing agent, muscarinic stimulation, lanthanides, Englerin-A and plasma membrane expression level of mutant channels are different from one study to another. We summarized the key findings regarding such issues in Table 4, from a number of reports, which we expect to be helpful to gain functional insight. 

### 5.2. Structure–Function Relationship in Cysteine Modification

Cryo-EM structure of mouse TRPC5 channel revealed that both C553 and C558 are located in S5-S6 linker. Likewise, C549 and C554 are also located in S5-S6 linker of TRPC4 channels. In both structural studies, authors not only revealed elegant Cryo-EM structures of the channels but also presented some critical electrophysiological recordings [51,52]. In TRPC4, extracellular DTT could not activate C549A and C554A mutant channels. The result would be probably due to the absence of active cysteines. When extracellular Englerin-A was applied, both C549A and C554A could not elicit current in response to the drug. Surprisingly, however, C549A/C554A double mutant elicited strong cationic current in response to Englerin-A. This result is somewhat in line with previously-mentioned C553S/C558S case [85]. In TRPC5, C553S, C558S, and C553S/C558S all failed to show significant current response to DTT or Englerin-A [51]. Based on the aforementioned structure and electrophysiological recording of TRPC4 channels, Duan et al. examined whether there exists any difference in S5-S6 linker sequence around two cysteine residues of TRPC4 and TRPC5 channels [51]. As a result, they have found that both channels share exactly same amino acid sequence between two cysteine residues, namely, ‘*C*KGIR*C*’. However, sequences upstream to first cysteine were different. TRPC4 has ‘ETKGLS’ sequence, while TRPC5 has ‘TRAIDEPNN’ sequence. Based on this speculation, they made chimeric TRPC5 channel whose S5-S6 linker sequence ‘TRAIDEPNN’ is changed to the one of TRPC4 (‘ETKGLS’) and examined how this chimeric TRPC5 channel respond to Englerin-A. In short, changing S5-S6 linker sequence could not make chimeric TRPC5 channel responsive to Englerin-A.

Structure gives clear location of key cysteine residues, namely, C549 and C554 in TRPC4 channels, and C553 and C558 in TRPC5 channels. Moreover, their location—S5-S6 linker—gives good explanation for their accumulated functional significance. Functional implication, however, looks way more entangled. It may be due to the cruel fact that cysteine modification is harnessed with various cellular mechanisms such as redox potential, tetramerization, ER-Golgi-plasma membrane trafficking process and protein folding.

## 6. G-Protein Mediated Gating Mechanism

### 6.1. General Aspect of G-Protein Mediated Gating Mechanism

As mentioned earlier in the article, G-protein coupled receptors (GPCRs), heterotrimeric G-proteins, and G-protein signal transduction pathways are one of the most classic activation mechanisms of TRPC channels. Among three most prominent subtypes of heterotrimeric G-proteins, namely, Gs, Gi and Gq, it was Gq-protein pathway and its implication to channel activation mechanism that have been studied most intensively. However, dissecting Gq pathway alone has been challenging. First, it is partly due to inherent complexity and dynamics within signal transduction pathway; one must consider all biomolecules participating in the signal transduction pathway—Gαq, Gβγ, PLC-β, PIP_2_, inositol 1,4,5-triphsophate (IP_3_), DAG, and PKC. Second, participants of signal transduction pathway are in delicate chemical equilibrium, hence up- or down-regulating one component immediately affects other components. For example, over-expressing gain-of-function mutant of Gαq may be useful to attest the effect of Gαq to TRPC channels. The interpretation becomes cumbersome, however, if at least one confounder participates in. For instance, if a finite amount of PIP_2_ was necessary to maintain activity of the channels, long-term effect of over-expressed Gαq would turn out to be negative to the channel activity regardless of its true nature. However, most of all, it was rapid and prominent desensitization of the channels that made dissecting downstream components out of overall effect of the Gq pathway to TRPC channels challenging. With an astonishing consistency, TRPC4 or 5 channels showed prominent desensitization right after strong activation whenever the channels were activated by Gq-protein coupled receptors (GqPCR, e.g., mAChR1, or 3, histamine receptor subtype 1) [19,21,22,37,38,39,40,41,42,43,59,71,72]. As an alternative, a non-specific G-protein activator such as [GTPγS]_i_ has been used [35,45,46]. Although GTPγS-induced channel current showed less or no desensitization, non-specificity of the drug still made the discrimination troublesome.

The development of Englerin-A made it possible to record G-protein independent, sustained TRPC4 and 5 currents [48]. Moreover, a recent advancement in biochemical tools must be also appreciated such as Gq proteins without PLC-β stimulatory effect, rapamycin-inducible systems, and voltage-sensitive phosphatases. Those methods all contributed to discriminate components of Gq downstream pathway and to measure individual effects to TRPC channels.

### 6.2. Diacylglycerol (DAG) and Protein Kinase C (PKC)

Earlier studies showed that only TRPC3 and TRPC6 are sensitive to DAG and can be directly activated by DAG or OAG [44]. Recent studies, however, showed that TRPC4 and 5 channels also have sensitivities to DAG and can be activated by it [86,87]. The pre-requisite must be sufficed, however, if TRPC4, 5 channels are to be activated by DAG. The channels elicited current in response to DAG only when phosphorylation of threonine at C-terminus (T972 of TRPC5) by PKC had been prevented. This claim was supported by the result that OAG elicited TRPC5 current only when PKC inhibitor was pretreated. TRPC5^T972A^ mutant channel showed cationic current in response to OAG. The study also correlated PKC phosphorylation to the PDZ-domain of TRPC4 and 5 channels. The existence of PDZ-domain (VTTRL) in C-terminus of TRPC4, 5 channels and their interaction with NHERF (Na^+^/H^+^ exchanger regulatory factor) protein have been already known. Since NHERF protein could serve as a scaffolding protein between cytoskeleton and any given protein containing PDZ-domain within, the verification of interaction per se between PDZ-domain containing TRPC channels and NHERF held much significance. The functional relevance for such interaction, however, was scarce. Using shRNA to NHERF proteins, the study has shown that TRPC5 current can be elicited by OAG when NHERF was down-regulated by shRNA.

The involvement of PKC in functional regulation of TRPC5 was also raised in another study but with different context [43]. What the study has found is that desensitization of carbachol-activated TRPC5 current was slowed as intracellular calcium increased. Thus, it was supposed that desensitization process may be calcium-dependent. They also have shown that desensitization process was slowed down when PKC inhibitors were treated. TRPC5^T972A^ mutation also yielded full activation but no desensitization under carbachol stimulation. The study concluded that desensitization process shown in GqPCR activated channels is partly due to PKC phosphorylation of the channel at T972 residue.

### 6.3. Phosphatidylinositol 4,5-bisphosphate (PIP_2_)

There are a number of issues surrounding PIP_2_ and activity of TRPC1, 4, and 5 channels. One of the earliest functional implications of PIP_2_ was addressed in the study that investigated alternative splicing variants of TRPC4 channels. Both human and mouse TRPC4 channels, like other proteins, do have natural splicing variants. One variant holds full exon sequence, hence called unspliced isoform or TRPC4α. The other variant has 84 less amino acids than the former and is called TRPC4β. Alternative splicing occurs at the C-terminal cytoplasmic domain of the channel (785–868th a.a. with respect to TRPC4α). Using over-expression system of two splicing variants in HEK293 cells, a study suggested that PIP_2_ exerts tonic suppressive effect to TRPC4α [42]. When diC-8, a water soluble analogue of PIP_2_ was added in intracellular solution, the GTPγS-induced whole-cell current of TRPC4α was reduced. On the other hand, the effect of PIP_2_ onto TRPC4β was minimal. Therefore, the authors concluded that 785–868 residue of TRPC4α is the discriminant for PIP_2_ governance to TRPC4 channels and that PIP_2_ may directly bind to the residue. Other phosphoinositides such as PI(3)P or PI(4)P showed no effect to TRPC4 channel, hence the effect can be seen as PIP_2_-specific. Despite of tonic suppressive effect of PIP_2_, PIP_2_ depletion alone could not activate both TRPC4 variants suggesting that the action of PIP_2_ has much meaning once the channel is opened by other activators.

Other studies claimed that PIP_2_ is involved in desensitization process of TRPC5 channels [41]. In HEK293 cells, the whole-cell current of over-expressed mouse TRPC5 channels was elicited by muscarinic receptor stimulation (mAChR_3_). Remark that stimulation of GqPCR strongly activates TRPC channel but prominent desensitization follows through the activation. In normal conditions, a mAChR_3_-activated TRPC5 current showed a similar time-course. When diC-8 was added in intracellular solution, however, desensitization was significantly slowed. The result implicated that desensitization can be partly attributed to decrease in PIP_2_.

More direct investigation to desensitization process and PIP_2_ level was achieved using rapamycin-inducible system. Rapamycin-inducible system utilizes rapamycin-dependent dimerization process of two proteins: FKBP (FK506-binding protein) and FRB (FKBP-rapamycin-binding domain). Namely, for two proteins A and B that can be fused with FKBP or FRB, spatial proximity between A and B can be achieved by making dimers out of FKBP and FRB. For instance, A-FKBP and FRB-B can form a dimer (A-FKBP:rapamycin:FRB-B) once the rapamycin is supplied in the system. The binding of rapamycin onto FKBP or FRB is noncovalent, hence reversible.

Like in TRPC5, carbachol (mAChR_3_) activated TRPC4β current measured in HEK293 cells showed strong activation followed by desensitization [39]. When PIP_2_ was added in intracellular solution, no desensitization of TRPC4β was observed. The study designed Lyn11-FRB and FKBP-Inp54p system where Lyn11 is a membrane-bound protein and Inp54p is an enzyme that can hydrolyze PIP_2_ into PI(4)P. In such a system, rapamycin would recruit Inp54p to the inner border of plasma membrane, which unless otherwise would be located diffusely in the cytosol. Unlike muscarinic stimulation, GTPγS-activated TRPC4β current shows slow activation but no prominent desensitization. What the study has found is that for Lyn11-FRB and FKBP-Inp54p expressing cell, a sudden desensitization process was observable in GTPγS-activated TRPC4β current as soon as rapamycin was applied. Moreover, the study also showed that the desensitization process of TRPC4β is independent from phosphorylation of the channel by PKC since the desensitization of the channel was still observable even in the presence of PKC inhibitor (chelerythrine, GF109203X). The desensitization was also retained in TRPC4β^T887A^ channel where T887 is equivalent site for T972 in TRPC5, hence putative PKC phosphorylation site of TRPC4β channel.

**Figure 5:**
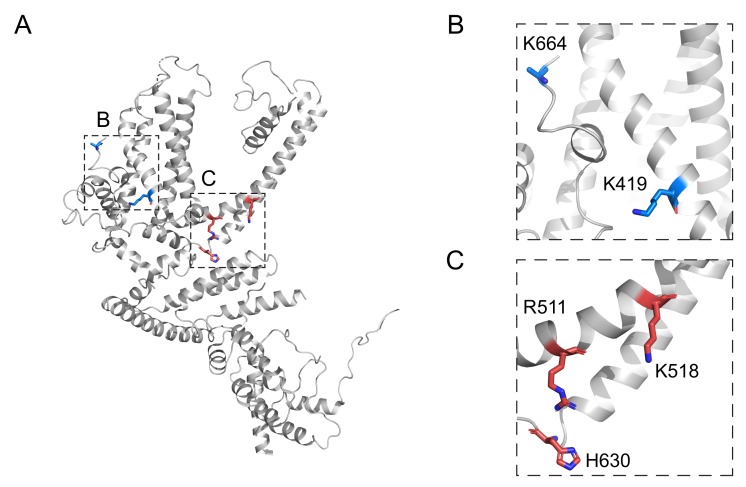
Putative binding pocket for PIP_2_ in TRPC4 channels. (**A**) Two binding pockets shown in one subunit of the channel. (**B**) First binding pocket consists of K664 and K419. (**C**) Second binding pocket consists of R551, K518, and H630.

A different study was reported which shares the similar context that PIP_2_ is necessary to maintain the opening of TRPC4β [37]. In this study a voltage-sensitive phosphatase (VSP) system was employed. VSP has an ability to hydrolyze PIP_2_ into PI(4)P and phosphate and the enzyme performance is increased once depolarized pulse is applied. The more positive and more frequent the pulse is, the more enzymatic reaction of PIP_2_ hydrolysis by VSP can be. Using the VSP system, the study has shown that the Englerin-A-activated current of TRPC4α, TRPC4β and TRPC5 channels were all inhibited by activation of VSP by depolarized pulse sequence. The study also monitored PIP_2_ level and whole-cell current simultaneously, calculating PIP_2_ sensitivity (IC_50_ value for I/Imax) of each channel. PIP_2_ level was monitored by FRET signal coming from PH-CFP and PH-YFP. PH domain is a subdomain of PLC-δ responsible for high affinity of PLC-δ to PIP_2_. The calculated K_d_ values for PIP_2_ showed following relationship: K_d_^C4β^ < K_d_^C4α^ < K_d_^C5^. The study postulated two putative binding pockets for PIP_2_ within TRPC4β channel as well based on a calculated structural model of TRPC4β using the Cryo-EM structures of TRPM4 and NOMPC channel at the time as the templates. K419, K664 constituted one binding pocket and R551, K518, H630 constituted the other pocket (Figure 5).

It has been also addressed whether PIP_2_ exerts regulatory effect to heteromeric TRPC channels. Using VSP and rapamycin-inducible system, one study showed that desensitization of TRPC1/4β and 1/5 channels shown in carbachol stimulation is due to PIP_2_ depletion [38]. The paper which measured K_d_ value for PIP_2_ inhibition also showed that desensitization of TRPC1/4α, TRPC1/4β, and TRPC1/5 are due to hydrolysis of PIP_2_. Moreover, they have shown that dispersed K_d_ values of TRPC4α, 4β and 5 channels converge to a singular value once TRPC1 is incorporated to the channels. In other words, K_d_ values of TRPC1/4α, TRPC1/4β, and TRPC1/5 were not different from one another.

A criticism was raised against the notion that desensitization process is solely dependent on PIP_2_. One study claimed that there exist additional pathways that must be considered to explain desensitization process of heteromeric TRPC1/4β and TRPC1/5 channels [88]. The study examined carbachol-activated TRPC1/4β, TRPC1/5 current in mAChR_3_-expressing HEK293 cells with and without PKC inhibitor. The desensitization was slowed down in TRPC1/5 when PKC inhibitor was pre-incubated but not in TRPC1/4β. They also examined carbachol-activated current of TRPC1/4b^T887A^ and TRPC1/5^T972A^. The desensitization of TRPC1/4β^T887A^ was not different from TRPC1/4β but TRPC1/5^T972A^ showed slower desensitization than TRPC1/5.

In summary, it seems clear that PIP_2_, a plasma membrane constituent, yields a substantial effect to the activity of TRPC channels. The alternative splicing of TRPC4 resulted in different sensitivity to PIP_2_ modulation. Accumulated results suggest that for a sustained opening of the channel, a finite amount of PIP_2_ near the channel is necessary. If the PIP_2_ level is decreased, activated channel seem to fall into inactivation. PIP_2_ alone, however, could not activate the channel, suggesting that the effect of PIP_2_ is meaningful only if the channel is opened by another activator. For this finding, the help of direct channel activator, Englerin-A, was paramount. If we apply the context to GqPCR-activated channels, activation of the channel must have been occurred before PIP_2_ hydrolysis begin to inactivate the channel. The Gq signal transduction pathway leaves no other choices for activation mechanism which is upstream to PIP_2_ hydrolysis: Gαq protein itself.

### 6.4. Direct Activation by Gαq Protein

Early studies questioning the possibility of direct TRPC channel activation by Gαq protein showed negative results [45,46]. It may be due to the lack of direct pharmacological agonist or antagonist for Gαq protein. The alternative experimental setup was to heterologously express gain-of-function mutant of Gαq proteins. Based on autonomic GTPase activity of alpha subunit of heterotrimeric G-proteins, a gain-of-function mutant of Gαq protein was generated. Once the glutamine residue crucial for autonomic GTPase activity is mutated to leucine, GTP bound form of alpha subunit, i.e., active Gαq could not hydrolyze the GTP, hence remain constitutively active. This Q to L mutation would surely augment the activity of Gαq protein but might be too strong to see adequate activation process of TRPC channels. In other words, even if Gαq could directly activate the TRPC channels, the overall effect of over-expression of Gαq ^QL^ protein would turn out negative since all of the PIP_2_ necessary for maintaining the TRPC activity would have been depleted.

The great breakthrough came out with the help of Gαq^QL/LA^ mutant. This mutant Gαq protein has constitutive activity due to QL mutation but cannot interact with PLC-β, a downstream effector molecule. In rapamycin-inducible system using Lyn11-FRB and FKBP- Gαq^QL^, GTPγS-activated TRPC4β current was gradually reduced right after rapamycin was introduced [39]. In Lyn11-FRB and FKBP-Gαq^QL/LA^, however, no change was observable. A similar system was conveyed to attest direct effect of Gαq protein onto TRPC1/4β and TRPC1/5 heteromers [38]. In short, rapamycin-induced Gαq^QL^ could not activate TRPC1/4β or TRPC1/5 channels but Gαq^QL/LA^ could. Moreover, the interaction between Gαq and TRPC channels was verified by Co-IP. The reason rapamycin-induced Gαq^QL^ could not activate the channels is that the positive effect of Gαq^QL^ to the channel—direct activation—and negative effect—PIP_2_ hydrolysis—occurs in a similar time-scale. This may be attributed to high a turn-over rate of PLC-β or low initial-value of PIP_2_ at least in micro-domain level. How fast rapamycin-induced dimerization of FKBP and FBR occurs would be also an important factor as well. Interestingly, multiplying the time-course of both PIP_2_ level in plasma membrane measured by YFP signal from PH-YFP and level of Gαq^QL^ docked to plasma membrane measured by RFP signal from Gαq^QL^-RFP yielded highly similar curve to time-course of whole-cell current.

### 6.5. Direct Activation by Gαi Protein

The very notion that G-proteins other than Gq-PLC pathway might activate TRPC channels was hard to accept at the time probably because there had been enormous studies supporting the latter pathway. The idea started from an astounding response of TRPC4 and 5 channels to pertussis toxin (PTX). PTX is one of the most potent and specific blockers of alpha subunit of inhibitory G-proteins (Gαi). Jeon et al. has found that both TRPC4β and TRPC5 channels elicit large currents when muscarinic acetylcholine receptor subtype 2 (mAChR_2_) was stimulated by carbachol [46]. When PTX was treated, however, mAChR_2_ could not elicit any current at all. They also have shown that gain-of-function mutant of Gαi proteins (Gαi^QL^) increases whole-cell current of both channels. This effect was also abolished by PTX. Later, a more comprehensive study further extended the idea by showing that Gαi_2_ and Gαi_3_ are potent, direct activators of TRPC4β and TRPC5 channels, respectively [45]. The study ruled out the involvement of beta-gamma subunits in Gαi-mediated activation process of the channels. Finally, the study suggested that K715 and R716 residue of TRPC4β are responsible for direct interaction between Gαi protein. The pathophysiological implication of such activation mechanism was studied in cultured hippocampal neurons [75]. Over-expression of Gαi_2_^QL^ and TRPC4β channel resulted in reduced neurite outgrowth of the neurons. The study claimed that this pathologic response is due to excessive calcium influx via highly activated TRPC4β channels and subsequent activation of calcium-calmodulin dependent protein kinase II (CaMKII) downstream pathways.

Although Gαi proteins strongly activated TRPC4β channels with direct interaction, they couldn’t activate TRPC1/4β heteromeric channels [59]. In HEK293 cells expressing both TRPC1 and TRPC4β channels, a large outwardly-rectifying (TRPC1/4β) current was elicited when mAChR_3_ stimulation was employed. On the other hand, only a minute amount of doubly-rectifying current (TRPC4β) was observable when mAChR_2_ was stimulated. Over-expression of Gαi_2_ protein also elicited only doubly-rectifying whole-cell currents. The difference in sensitivity to muscarinic governance (mAChR_2_ or mAChR_3_) between TRPC4/4 and TRPC1/4 channels may explain TRPC4/4-dominance in ileal myocyte where mAChR_2_ governance was shown to be more dominant than mAChR_3_ [20,22].

### 6.6. Structure–Function Relationship in G-Protein Mediated Gating Process

**Figure 6:**
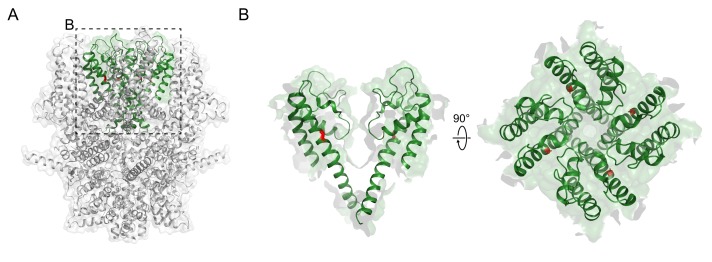
The homologous position of TRPC4 to G652 of TRPC3. (**A**) Glycine residue in tetrameric structure. *Green domain*: pore helices; *Red sticks*: glycine residues. (**B**) *Left panel*: enlarged view of pore structure and glycine. Subunits behind and in front of the paper is deliberately deleted for viewer’s preference. *Right panel*: top-view of pore-helices and glycine residue.

First, DAG-mediated regulation requires a corresponding lipid-binding pocket in the channel structure. For example, TRPV and TRPML, which are all activated by lipids—capsaicin and PI(3,5)P2—show binding pockets for their agonistic lipids [89,90]. Although Cryo-EM structures show some lipid binding pockets in both TRPC4 and TRPC5, conformation rejects the likelihood of the pocket being DAG-specific. Even the structure of TRPC3 could not represent DAG-binding pockets despite long-history for DAG-mediated gating mechanism of the channel [55,56]. One study, however, proposed a promising result [91]. Using a modeled structure of TRPC3 with TRPV1 as a template, putative DAG binding domain (G652) was postulated. Since sequence homology around the residue is high among TRPC channels, we have marked identical glycine residue in TRPC4 channels (Figure 6). Second, two residues bear special importance in terms of PKC-mediated regulation of the channels: T887 and T972. These residues are located in FSD and missing in Cryo-EM structure of both TRPC4 and TRPC5.

PIP_2_ holds exceptional position in Gq-mediated gating mechanism. In addition to functional implication in channel gating mechanism, a physiological relevance is also immense. By the virtue of calcium influx and membrane excitation, it carries substantial pathophysiological meaning whether the channel opens in episodic manner due to inherent desensitization or the opened channel sustains the opening or the channel stays refractory even in the presence of activators.

In spite of studies mentioned above, domain-specific functional studies against PIP_2_-mediated regulation of TRPC channels are still limited. K419, K664, and R511, K518, H630 are putative binding sites at least with functional evidence. The difference in PIP_2_ selectivity between homomeric channels and heteromeric channels may indicate that binding site for PIP_2_ might be overlapped with subunit-subunit interface. Provided that heteromeric subunits locate at *cis*-position one another rather than *trans*-position, PIP_2_ binding may require cooperative action of different residues in *cis*-positioned subunits.

In addition to the report that TRPC4α receives tonic suppression by PIP_2_ but not TRPC4β, a number of studies also presented results supporting that there exists a difference in characteristics between two splicing variants. Thus, a structural explanation for such event seems imperative. Unfortunately, however, the 84 amino acids are also included in FSD, hence no structural evidence can be provided for now.

In our best knowledge, no study has been addressed Gαq-binding site within TRPC1, 4, 5 channels so far. Putative Gαi-binding sites, however, were presented: K715 and R716. Cryo-EM locates both residues in the connecting-helix. 

## 7. Englerin-A Mediated Gating Mechanism

Englerin-A opens TRPC4/4, 5/5, 1/4 and 1/5 channels in extracellular side. Nanomolar concentration of Englerin-A is sufficient to activate the channels. As previously mentioned, there were no agonists with high specificity to TRPC1, 4, 5 channels. The development of this pharmacological treasure bestowed great utility for channel research especially in discrimination of G-protein signal transduction pathway. Depending on literatures, maximum conductance was reached within 10 to 20 s after Englerin-A was applied. This suggests that the drug action could be direct to the channel. Although it is unlikely, whether the increase of conductance in whole-cell current level is due to increased surface level or not is unknown. In outside-out patch, the increase in single channel conductance by Englerin-A was reported [48]. Nonetheless, domain-specific study for mechanism of action of Englerin-A is still limited.

One study addressed possible interaction sites of TRPC5 channels with Englerin-A [85]. Among the lists of mutants targeted for acid-based sensing domain of the channel, Jeong et al. found that K554, H594, and E598 of TRPC5 are putative binding sites for Englerin-A [85]. These residues are all located in pore-helices. From the proximity per se, Englerin-A may open the channel by changing conformation of the upper gate near selectivity filter.

**Figure 7:**
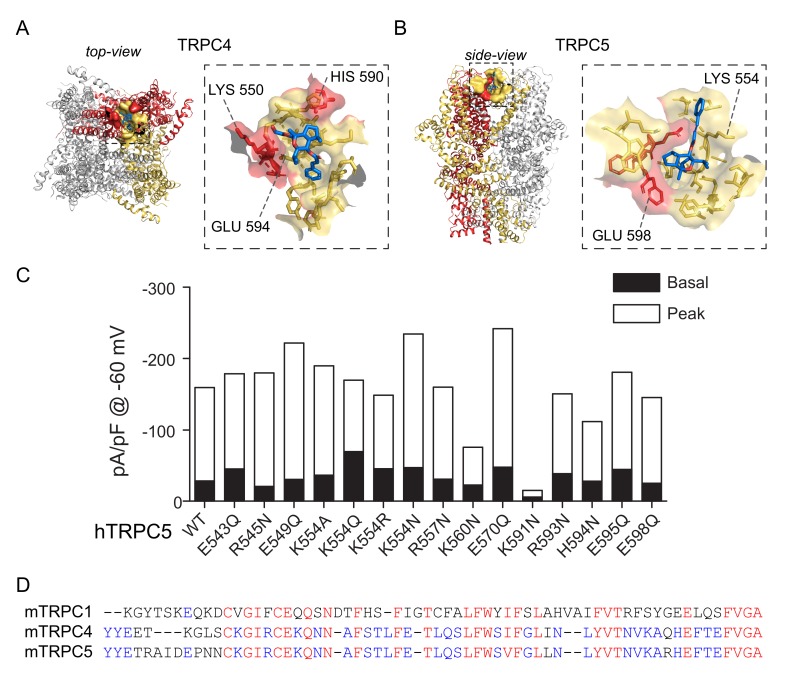
Binding models between Englerin-A and TRPC4, 5 channels. (**A**) Binding model for TRPC4. (**B**) Binding model for TRPC5. The model claims that not only the hydrogen bonding between hydroxyl group and polar residues but hydrophobic interaction is important in both cases. Yellow sticks, surfaces: subunit A; Red sticks, surfaces: subunit B; Blue sticks: Englerin-A (**C**) Electrophysiological recordings of TRPC5 with various point-mutations. Basal current of TRPC5 (black bar) and peak current (white bar) after extracellular application of 100 nM Englerin-A. (**D**) Sequence alignment of TRPC1, 4, and 5 channels along pore regions. TRPC1 shows significant difference while the TRPC4 and 5 channel share great similarity.

Based on previously reported putative Englerin-A binding sites, we calculated possible binding models. The Q386-H394 region had to be constructed since Cryo-EM structure of TRPC5 lacks such residues. Besides K554, K594, and E598, the additional constraint to the simulation was applied that extracellular S1-S2 linker and S3-S4 linker has nonzero likelihood of Englerin-A binding. As a primitive result, the model predicted that both in TRPC4 and TRPC5 there is a strong cooperativity between *cis-*positioned subunits. Therefore, we added additional constraint to the model that Englerin-A binds to TRPC4 channel via K550 in A subunit and H590, E594 in B subunit. Likewise, K554 in B subunit and H594, E598 in A subunit was selected as binding sites for TRPC5. The model suggests that the way TRPC4 and TRPC5 channels bind with Englerin-A is different (Figure 7). It also vests great significance to hydrophobic interaction (V, F, A, Y) in addition to acid-base sensing residues: K, H, and E. Last but not least, the prediction index showed different amino acids for highest weighting in TRPC1. The sequence alignment along the pore-region shows significant difference between TRPC4, 5, and TRPC1. Therefore, binding mechanism could be different in heteromeric channel and in homomeric channel. Structure of TRPC1/4 or 1/5 heteromeric channel would clarify the issue.

## 8. Concluding Remarks

Structural revelation of TRPC channels has clarified the issues or the controversies against the function of the channels. Still, there are a few challenging problems that need to be solved. One of the limitations is that given structures all show only closed state of the channels. We do not know yet how exactly the selectivity filter of the channel exerts its action to conducting ions. It is obscure which residues within the pore are responsible for stabilization of dehydrated or hydrated conducting ions. Neither the mean occupancy number of conducting ions nor whether the pore profile is multi-ionic or not is known. Hopefully, the exact roadmap for conformational change of the gates could be addressed in a near future. Structural analysis of the inward-rectification mechanism of TRPC4 and 5 channels will also be intriguing. Crystal structure of Mg^2+^-bound and polyamine-bound inwardly-rectifying potassium channels could be models for the study [92].

In terms of tetramerization, solving Cryo-EM structure of TRPC1/4 and TRPC1/5 channels has substantial physiological meaning since incorporation of TRPC1 to TRPC4 and TRPC5 yields reduced membrane excitability and calcium permeability [93]. Identifying the time and location of heteromerization process is rather hard to answer with structural methods. Additional functional studies targeted for protein trafficking process could show promising results.

As we have discussed, cysteine residues in the S5-S6 linker hold a number of issues. Finding consistent explanation for inconsistent results would be challenging, indeed. Opening of the channels by altering cysteines using extracellular reducing agent may constitute an independent gating mechanism aside from gating through the G-protein pathway or Englerin-A. It is imperative to solve a structure for channels opened by cysteine modification.

The most classical activation mechanism of TRPC channels would be undoubtedly a G-protein mediated gating. The emerging role of DAG in such gating process requires DAG binding pocket within not only TRPC3 and 6 channels but also in TRPC4 and 5 channels. The putative binding site predicted by modeling accompanied meaningful electrophysiological results, hence could be dearly appreciated. The G602 site may give a useful initial-value for the equation. What makes it hard to find structure–function relationship in G-protein gating mechanism is that the quintessential domains such as the PDZ-domain, alternative splicing site, and PKC phosphorylation site are all located in the far-south domain. A structural flexibility is a foe to Cryo-EM, hence truncation of flexible domains is usual prerequisite in sample preparation step. As a functional aspect, however, flexibility of domain may reflect functional significance. It is difficult to picture a key domain for gating in an immovable position provided that opening and closing of the channel is a highly dynamic process. Nonetheless, structures with minimal information of FSD would be greatly helpful to elaborate G-protein mediated channel gating process.

Finding PIP_2_ binding site within the channel would be equally important as to understand the channel function and for pathophysiological implication. Disturbance in PIP_2_ governance to TRPC channels could result in catastrophic collapse in intracellular calcium homeostasis since PIP_2_ depletion seems to be an effective negative feedback to direct activation of Gαq-protein. In a sense, a search for binding site to Gαq-protein within the channels is necessary. Structures for Gαi-bound TRPC4 or TRPC5 might be found faster than Gαq since putative binding sites are already known. Although it was X-ray crystallography that solved the structure rather than Cryo-EM, a study of Gβγ-bound GIRK (G-protein activated Inwardly-Rectifying K channel) could be viewed as an excellent reference [94].

The development of Englerin-A has delivered unprecedented pharmacological utility. A lot of studies analyzed functional consequences of ‘Englerin-A activated’ TRPC channels in numerous experimental conditions. It also implies that such outcomes are not free of confounding-bias unless the exact mechanism of the drug is addressed. We believe that fast evolving Cryo-EM will bear a fruit in the near future and that mechanistic study for Englerin-A in parallel will definitely accelerate the pace.

## Figures and Tables

**Table 1:** Result of site-directed mutagenesis targeted for residues in ion conduction pathway.

Residues	Gd^3+^	Englerin-A
N584A	-	+, minute activation
G581A	-	+++, fast inactivation
I621A/N625A/Q629A	-	+++, slow inactivation

**Table 2:** Summary of domains responsible for homo- or hetero-tetramerization of TRPC 4 channels.

	Region (Residue)	Reference
1	2nd and 3rd ARD (87–172)	[77]
2	3rd ARD (98–124)	[78]
3	HLH (254–304)	[77]
4	Connecting Helix (700–728)	[78,80]

**Table 3:** Summary of domains responsible for homo- or heterotetramerization of TRPC 5 channels.

	Region (Residue)	Reference
1	2nd ARD (69–98)	[76]
2	Connecting Helix (707–735)	[80]
3	C553	[79]

**Table 4:** Summary for accumulated results investigating outcome of cysteine modification.

		Current Response to				Surface Level
		La^3+^ or Gd^3+^	Englerin-A	Reducing Agent	Muscarinic Stimulation	
TRPC5	C553X	Reduced [51] Reduced [79]	Reduced [51]	Reduced [51] Reduced [79]	Reduced [79] Reduced * [83]	Reduced [51] Reduced [79] Intact [83]
	C558X	Reduced [51] Reduced [79]	Reduced [51]	Reduced [51] Reduced [79]	Reduced [79] Reduced * [83]	Reduced [51] Reduced ^†^ [79] Intact [83]
	C553X/C558X	Reduced [51] Reduced [79]	Reduced [51] Intact [85]	Reduced [51] Reduced [79]	Reduced [79] Reduced * [83] Reduced ^‡^ [85]	Reduced [51] Reduced [79] Intact [83]
TRPC4	C549X	N/A	Reduced [53]	Reduced [53]	N/A	N/A
	C554X	N/A	Reduced [53]	Reduced [53]	N/A	N/A
	C549X/C554X	N/A	Intact [53]	Reduced [53]	N/A	N/A

N/A: Not addressed, X: alanine or serine; * Channel activity measured by Ca^2+^ indicator and F_340_/F_380_ but not current; ^†^ Authors specifically mentioned in the article that only minute amount of surface level was reduced; ^‡^ overexpression of gain-of-function mutant of Gα_i2_.
